# Salt-Free Pickling with Sulfonic Acid as an Approach to Cleaner Leather Processing

**DOI:** 10.3390/ma19030471

**Published:** 2026-01-24

**Authors:** Renata Biškauskaitė-Ulinskė, Virgilijus Valeika

**Affiliations:** Faculty of Chemical Technology, Kaunas University of Technology, Radvilenu pl. 19, 50254 Kaunas, Lithuania; renata.biskauskaite@gmail.com

**Keywords:** hide, leather, pickling, *p*-toluenesulfonic acid monohydrate, chrome tanning

## Abstract

Recently, increasing attention has been paid to the application of sulfonic acids as alternative materials for the pickling process. The aim of the present study was to investigate the action of pickling with *p*-toluenesulfonic acid monohydrate on derma’s collagen and the influence of this action on subsequent processes and properties of chromed and crust leather. The application of *p*-toluenesulfonic acid monohydrate in the pickling process led to a similar effect on collagen compared with conventional process. The solutions after experimental pickling contained lower amounts of total dissolved solids, total suspended solids, and chlorides. The chrome tanning process is improved after the pickling with *p*-toluenesulfonic acid monohydrate: the exhaustion of chromium compounds reaches 98%, while after conventional pickling, it is only 68.7%; accordingly, lower amounts of basic chromium sulfate can be used for chrome tanning to achieve the same chromium content in the wet blue leather. The crust leather produced after experimental pickling has properties close to the conventional one.

## 1. Introduction

Leather processing technologies involve a series of complex processes that purposefully alter the structure of the skin’s/hide’s dermis and remove substances that are undesirable in terms of leather quality. Usually, the main role here is played by the processes prior to tanning [1]. Tanning is the key process after which the resulting product, characterized by thermostability and resistance to the effects of enzymes and microorganisms, is considered to be processed leather [2].

To date, more than 90% of leather is processed using chrome tanning [3]. This type of tanning is relatively simple and convenient to perform from a technological standpoint. On the other hand, the use of chromium compounds is considered unfavorable from an environmental perspective, as it represents one of the most polluting operations in the leather-making process [4]. In European countries, EU directives for the leather industry have limited the use of chrome tanning, making it important to establish new techniques and strategies to ensure environmental protection [5].

The pickling process is primarily conducted to adjust the collagen to the conditions required by the chrome or any other tanning [6]. The purpose of this process is to significantly reduce the pH of the hide before chrome tanning, to make it permeable to tanning compounds and to prevent it from swelling. The acid absorbed during pickling changes the basicity of the chrome tanning compounds during tanning and regulates the chromium tanning process. A mixture of sulfur, acetic acid or formic acid, and sodium chloride is most commonly used for pickling. Using this solution changes the pH value of the hide, reduces swelling, and prepares the hide for chrome tanning [6].

Although pickling methods are relatively well established, there are still some significant shortcomings, which have led to exploring the alternative and more effective ways to prepare hides for tanning:

1. Insufficient consumption of chromium compounds after conventional pickling, which, according to various literature data, ranges from 55 to 75% [7,8]. This means that almost half of the chromium used ends up in wastewater [8].
2. The conventional pickling is particularly problematic due to the enormous amounts of chloride emissions, which have been a longstanding issue that has not been satisfactorily resolved [8]. The process requires about 7–10% sodium chloride on processed skin/hide mass [9,10]. Data presented in the Compendium of the Food and Agriculture Organization of the United Nations [11] shows that during 2015, about 6.3 billion tons of hides/skins (generally bovine, sheep, lamb, and goat hides/skins) were processed into leather. Accordingly, about 0.63–0.44 billion tons of sodium chloride are necessary annually only for the pickling, and all this eventually falls into wastewater.

A review of research conducted in this field over the past two decades has revealed the following key trends in pickling improvement.

The first is an addition of auxiliary materials to conventional pickling to promote better consumption of chromium compounds during chrome tanning. Yao et al. report that carboxyl-terminated hyper-branched polyester (HPB) is extensively used as high-exhaustion chrome-tanning auxiliary. The use of HBP can endow the wet blue leather with up to 95.2% chrome uptake, much higher than the control trial (69.3%) [12]. Continuing this research, an amino-terminated hyper-branched polyamide (HBPN) was synthesized and used for pickling pigskin: incorporation of 1% HBPN into the pickling process significantly improved chrome uptake to 83.32% and increased the hydrothermal stability of leather [13].

Jia et al. [14] synthesized an amphoteric polymer (AP) produced by a reaction between dimethyl diallyl ammonium chloride, acrylic acid, and sodium allyl sulfonate and added the polymer into pickling, replacing some sodium chloride. The use of AP polymer increased the Cr_2_O_3_ content in the leather by 10.8% and improved the chromium distribution compared with conventional leather. Other properties of the leather treated with AP were close to those of the conventional leather.

Another study using tetrakishydroxymethylphosphonium sulfate showed an enhancement of chromium in the leather, due to the material’s tanning effect, and using it improved the uptake of chromium compounds during chrome tanning [15].

The next option for pickling development was to eliminate the use of sodium chloride. Li et al. demonstrated that oxazolidine is a potential material to replace neutral salts in pickling and can achieve high chrome exhaustion in subsequent chrome tanning [16]. To reduce the environmental impacts of chrome tanning, a salt-free chromium tanning technology has been developed and optimized based on the combined use of polyoxyethylene diepoxy ether and urotropine. The results indicated that, without the application of sodium chloride, the hides could be protected from acidic swelling in pickling and chrome tanning, and the chromium uptake rate increased from 74.3% to 96.0% [17].

Some researchers have attempted to replace pickling by treatment with other materials or omit this processing step altogether. Rao et al. [18] adopted a polymeric syntan, which can enable pickle-free chrome tanning. The method enhances the chromium uptake to above 90% and reduces the chemical oxygen demand (COD), total dissolved solids (TDSs), and chlorides in the spent tan liquor. To replace the traditional pickling process, an advanced polymeric material based on acrylic acid has been used [19]. The application of the polymeric processing intensifies the tanning process and increases the thermostability of leather.

Naisini et al. [20] suggested a chemical mixture of sulfuric acid and basic chromium sulfate, which leads to pickle and basification-free single-pot chromium tanning. The process reduces effluent load generation, exhibiting nearly 99% chromium uptake, while the conventional one is only about 69%; there is more than a 99% reduction in TDS, chlorides, and the COD load [21].

Another important consideration in pickling development is the reuse of pickling wastewater after electrochemical treatment. Treated wastewater was reused, and the cycle was repeated seven times [22].

All the mentioned methods require additional chemical materials, such as new polymers or specially prepared chromium compounds, or involve processes like electrochemical treatment. On top of that, managing the order of the processes becomes much more complicated.

In this regard, the use of sulfonic acids or their derivatives to completely or partially replace the materials that are commonly used for pickling is deemed promising. Haroun and Ahmed [7] write that aromatic sulfonic acids can form an electrovalent bond with the amino groups on the collagen side chain, conceal the positive charges on the side chain of collagen molecules, and prevent possible acidic swelling of hides in the absence of sodium chloride. Naphthalene sulfonic acid and sulfosalicylic acids were applied and the chromium absorption rate increased to 99% compared to 75% in the conventional process. Yang et al. [23] applied 5-sulfosalicylic acid, 1,5-naphthalenedisulfonic acid, and 2-naphthalenesulfonic acid instead of conventional pickling materials and concluded that the adsorption rate of chrome-tanning agent to the hide surface was increased. The properties of wet blue leathers and crust leathers pickled with aromatic sulfonic acids were inferior to those of wet blue and crust leathers pickled conventionally.

Sulfosalicylic acid, naphthalene sulfonic acid, naphthol sulfonic acid, and phenol sulfone sulfonic acid were explored as materials for pickling [24], and it was established that pickling with naphthalene sulfonic acid under salt-free conditions did not cause acidic swelling of the hides or over-pickling and increased chrome uptake in the novel process up to approximately 98%. Zhang et al. [25] synthesized three aromatic sulfonic acids and evaluated them as pickling agents for hide processing, replacing sulfuric acid and sodium chloride. The results showed that the novel salt-free pickling produced clean pores and well-dispersed fibrils. Also, it improved the chromium absorption and distribution in crust leather from 71.6% to 98.6%.

The data presented indicate that sulfonic acids are promising materials and can be used in leather processing. *p*-toluenesulfonic acid monohydrate (*p*TsOH) is a strong acid and oxidizer, and therefore acts accordingly; however, it is not classified as a carcinogenic, mutagenic, or teratogenic material or reproductive toxicant [26]. Accordingly, it can be adopted for leather processing as any other acid or oxidizer used in tanneries.

The aim of this research was to investigate the suitability of *p*-toluenesulfonic acid monohydrate as an alternative to conventional pickling materials and to establish the effect of the acid on pickling, chrome tanning, and chromed and crust leather properties.

## 2. Materials and Methods

### 2.1. Materials

For the studies, salted cattle hide was used as a raw material (purchased from TDL Oda Ltd., Šiauliai, Lithuania). For the experiments, the hide was cut into 5–10 × 8–15 cm pieces, which were asymmetrically divided into experimental series so that each series contained samples from all the topographic areas of the hide.

The chemicals used for the analysis were of analytical grade. Analytical- and technical-grade materials were used for the technological processes.

Other technical products used for the technological processes were the following: Cromeco 33 Extra (contains 25% of chromium (III) oxide, 33% basicity) produced by Gruppo Chimico Dalton (Limbiate, Italy); Neutragene MG-120 for increasing the chromium compounds’ basicity (Codyeco S.p.a., Santa Croce sull’Arno, Italy); fatliqours Oleal 146, Oleal 1946, Fospholiker 661 and Fospholiker 6146 (Codyeco S.p.a., Italy); dye Sellaset red H (TFL Ledertechnik GmbH, Rheinfelden, Germany); and mimosa tannins and quebracho tannins (Tanac S.A., Montenegro, Brasil).

### 2.2. Technological Processes

All technological processes were carried out as follows (Table 1).

### 2.3. Analysis of the Solutions

After the process solutions were analyzed, various parameters were identified.

The amount of collagen protein removed was estimated by quantifying the amount of hydroxyproline in the solution using a photo-colorimetric method [27]. Samples of the pickling solution collected after the process were hydrolyzed with 6 N HCl at 120 °C for 10–12 h. The formation of a colored, soluble product was based on the reaction of hydroxyproline with p-dimethylaminobenzaldehyde. Absorbance was measured at 558 nm using a GENESYS-8 spectrophotometer (Spectronic Instruments, Cheshire, UK).

The amount of non-collagen proteins removed to the solution was determined using the Lowry method [28]. The assay relies on a colorimetric reaction in which the protein interacts with an alkaline copper tartrate solution and Folin reagent. The absorption of the blue-colored reaction product was measured with a spectrophotometer at a wavelength of 660 nm.

The NaCl concentration in the solution was determined by titration with 0.1 mol/L HgNO_3_ [29]. Mercuric and chloride ions form a highly stable soluble complex; excess mercuric ions react with diphenyl carbazone (indicator) to form a blue–violet complex.

The concentration of chromium in solution was determined according to the method described in the literature [30]. The method prescribes oxidation of the chromium presented in the solution into a hexavalent state using hydrogen peroxide, and analysis of the solution by iodometric titration.

The exhaustion of the dye was quantified using the colorimetric method by measuring the absorbance of the dye solution. The dye absorbance was measured at 495 nm using GENESYS-8.

The TDS and total suspended solid (TSS) concentrations were determined as described in the literature [31]. The TDS is determined by filtering a well-mixed solution sample through a membrane filter (pores—0.45 µm; initially determined constant weight after heating at 105 ± 1 °C) into a weighted dish; evaporating the filtrate; and drying to a constant weight at 180 ± 1 °C. The increase in weight of the dish corresponds to the amount of TDS. The membrane filter with solids is dried to a constant mass at 105 ± 1 °C, and the increase in the weight of the membrane filter corresponds to the amount of TSS.

The pH of the solutions was measured using pH-meter pH-526 (WTW, Bremen, Germany).

### 2.4. Analysis of Hide and Wet Blue

The pieces from the butt part of hide in the experimental series were marked and later used for the determination of qualitative parameters of hide and leather as prescribed by the standard [32]. The shrinkage temperature following beam-house operations and post-tanning processes was determined using specialized equipment, as described in the literature [33]. The test uses water or a mixture of glycerol and water (depending on the potential hide shrinkage temperature—up to or above 100 °C).

Hide porosity was measured using 4 × 4 cm acetone-fixed hide samples. Each sample was weighed, submerged in kerosene (at a height three times the leather’s thickness), and placed in a vacuum desiccator for 5 min. After removal, excess kerosene was blotted away, and the sample was reweighed. The difference in mass was equal to the mass of kerosene that has penetrated the pores of the sample under vacuum. The average porosity of the sample was calculated as a percentage using the sample volume and the density of kerosene (0.794 g/cm^3^).

(1) $$P=\frac{m_{2}-m_{1}}{d\cdot V}\cdot 100;$$

*m*_2_—mass of hide/leather sample after adsorbtion of kerosene, g;

*m*_1_—initial mass of hide/leather sample, g;

*d*—density of kerosene, g/cm^3^;

*V—*volume of hide/leather sample cm^3^.

Infrared spectroscopy (FTIR) analysis was carried out for samples after pickling and chrome tanning using a Perkin-Elmer FTIR Spectrum GX (Waltham, MA, USA) spectrometer with a horizontal attenuated total reflectance accessory. The scan number was 10 times, resolution—4 cm^−1^. The wavelength interval was 4000–650 cm^−1^. Before FTIR analysis, 1 mm of dehydrated sample’s grain layer was split and IR spectra recorded from the obtained surface.

The optical microscope images were obtained, and the penetration of dye through the hide was determined using a microscope with scale (magnification 15 times) MPB-2 (Izyum Instrument Making Plant, Izyum, Ukraine). The dye penetration was calculated by estimating the width of the cross-section of the dyed and undyed leather.

The strength properties, amount of chrome compounds in the leather, matter soluble in dichloromethane, and hide and chromed leather pH values were determined according to the standards [34,35,36,37].

Before porosity, FTIR analysis, and mechanical tests, hide and wet blue leather were treated with acetone to fix their structure [30] and conditioned for 24 h at a temperature of 23 °C and a humidity of 52%.

### 2.5. Statistical Analysis

All data were expressed as the average value of measurements performed in triplicate. One sample was used for one measurement. Standard deviations did not exceed 5% for the values obtained.

## 3. Results and Discussion

To evaluate the potential application of *p*TsOH in leather processing, the suitability of this acid in the pickling process was first investigated by replacing commonly used materials such as NaCl, NaCOOH, and H_2_SO_4_ and reducing the pickling time by 1 h and 15 min. Salt-preserved cattle hide samples were treated together until pickling according to the technology presented in Table 1. After bating and washing, the samples were divided into seven groups, and six of them were pickled using *p*TsOH (samples of the seventh group were pickled conventionally), which was added at a total amount of 0.5–3% (% of the mass of the hide). All pickling parameters are presented in Table 2.

After pickling, the condition of the hide was first assessed organoleptically: swelling, damage to the grain layer of the sample, softness, as well as the shrinkage temperature and pH of the pickling solution were evaluated. Both the shrinkage temperature and pH are important parameters, as the change in shrinkage temperature can be used to assess the effect on collagen, and the pH of the solution at the end of the process is important for the subsequent chrome-tanning process. Chrome tanning is usually carried out in a pickling solution, in an acidic medium with an initial pH of 2.0–3.0. In such a medium, most of the carboxyl groups of pickled collagen are protonated and interact only weakly with Cr^3+^ ions, thereby allowing the chromium compounds to penetrate deeper into the derma [38]. An excessively high pH of the solution may cause chromium compounds to fix on the surface [24].

During pickling, the pH was measured several times to see the range of pH changes in the pickling medium (Table 3). The results show that at the beginning of the process, the lowest pH was obtained when conventionally pickled, i.e., using sulfuric acid, but at the end of the process, the lowest pH was obtained when pickling with 3% *p*TsOH—the pH of the solution was 2.1. As can be seen from the results, 0.5% and 1% *p*TsOH are not sufficient for the pickling process, as the pH of the solutions obtained at the end of pickling was as high as 7.87 and 7.27. When chrome tanning is performed in such a solution, it is likely that chromium compounds will be fixed on the surface of the leather, as the carboxyl groups of the collagen in the hair will not be protonated [38]. The higher pH values obtained by adding 0.5% and 1% *p*TsOH can be explained by the fact that after liming and bating, the pH of the hide was 8–9, and this amount of acid was sufficient only for neutralization; a larger amount of acid was needed to reduce the pH of the hide.

After pickling, the samples were removed from the solutions, and the softness or hardness of the hide was evaluated organoleptically. When comparing the softness of the samples, the first two experimental variants with low *p*TsOH concentrations were much harder than the other samples. Organoleptic testing showed that the softness of the samples pickled with 1.5% *p*TsOH was similar to that of the control sample, while higher concentrations resulted in softer samples. The softest sample was the one treated with 3% *p*TsOH, but the hide was slightly swollen, and its elasticity was reduced (when pressed with a finger, the sample did not return to its original state and a mark remained). A slight shrinkage on the surface was also observed when using 2.5% *p*TsOH.

The results of the hide shrinkage temperature after pickling show that all experimental samples, except those treated with 3% *p*TsOH, had a higher shrinkage temperature than the control sample (Table 4). This shows that the use of 0.5–2.5% organic acid had a weak effect on the hide, and the collagen was more thermally stable after such experimental pickling than after being conventionally treated. The hide pickled with 3% *p*TsOH had the lowest shrinkage temperature of 51.3 °C and the lowest pH of 1.93. The pH of the pickling solution (2.10) was also lower than after control pickling (2.24), possibly because the 3% acid concentration was too high. This was also confirmed by organoleptic evaluation—the semi-finished product was characterized by severe swelling and reduced elasticity due to possible acid swelling during pickling. Unlike alkaline swelling, acid swelling irreversibly destroys the collagen structure, and due to this, must be avoided [39].

After the results of the shrinkage temperature and organoleptic evaluation, it was decided not to proceed with further studies using 2.5% (observed shrinkage of the outer layer) or 3% *p*TsOH. Further studies were conducted on pickling using 0.5–2% *p*TsOH (based on the hide mass).

With an increasing organic acid concentration, the shrinkage temperature of the pickled samples decreased, suggesting that a higher acid content had a stronger effect on the hide. At higher concentrations, collagen may have interacted more extensively with the acid. The amount of collagen proteins removed in the solutions after pickling did not show a clear dependence on the concentration of the acid used; however, more collagen proteins were removed during the experimental process than during the conventional method. Control pickling resulted in a higher hide porosity, as well as lower collagen protein removal.

Pickling results indicated that when less (0.5–1.5%) *p*TsOH was added, the pH of the hide remained considerably higher compared to other variants. If chrome tanning is performed after pickling, when the pH of the pickled hides is 6.20–7.39, it is likely that chromium compounds will be fixed on the surface layers of the hide, as chromium compounds react with collagen in these layers. The former will penetrate the inside of the hide with difficulty, which might determine the hardening of the semi-finished product [24].

**Figure 1:**
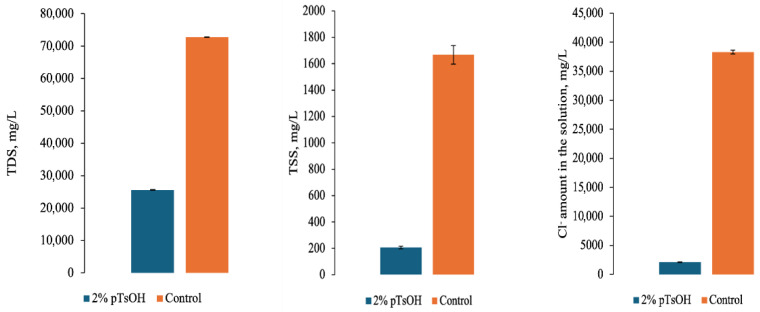
TDS, TSS, and Cl^-^ amount in pickling solution, mg/L.

Pickling with 2% *p*TsOH showed the greatest potential. The pH of the pickling solution obtained after the process was more suitable for further chrome tanning than when using lower acid concentrations, also swelling or shrinkage of the surface was not observed. Prior to chrome tanning, pickling was carried out again, and TDS, TSS, and chlorides in wastewater were determined (Figure 1).

The results showed that after the experimental process, the concentration of TDS in the wastewater was lower than after conventional pickling; in the latter case, there were more than 2.8 times more dissolved solids than after treatment with *p*TsOH. Furthermore, after determining TSS in the solutions, it was found that pickling with *p*TsOH had an advantage, as there were eight times fewer such particles than when using NaCl and sulfuric acid. Lastly, chlorides in the effluent were also analyzed.

Normally, during pickling, a large amount of chlorides remains in the pickling solutions. In the experiment solution, even though no NaCl was added during pickling with *p*TsOH, chlorides were determined as well. This might be due to the fact that chlorides can enter the solution from the treated hide as well as from the tap water, as it was used during the experiments to imitate industrial leather processing. The results of Cl^-^ in effluents show that pickling with *p*TsOH had advantages over conventional pickling in terms of chloride pollution. The chloride concentration in the experimental wastewater was more than 18 times lower than after conventional pickling. Accordingly, such wastewater would be easier to treat further than after conventional pickling due to the lower chloride content.

To evaluate the possible effect of the pickling on the hide structure changes, an FTIR analysis was performed. The IR spectra were recorded and analyzed (Figure S1). The analysis of both spectra did not show any differences. This allows the conclusion that pickling with *p*TsOH does not cause observable additional chemical changes in hide structure compared with the conventionally pickled hide.

After evaluating qualitative parameters of pickling, chrome tanning was performed in the pickling solution. The following indicators were determined to assess both the process and the resulting semi-finished product: pH values of the solution and the wet blue, shrinkage temperature, and chromium compound exhaustion (Table 5). Pickling was carried out in two ways: using the conventional pickling method (control) or by adding 2% *p*TsOH to the pickling solution, followed by chrome tanning according to the method presented in Table 1.

The results obtained after chrome tanning show that 2% *p*TsOH was the appropriate amount for the pickling process. The tendency of a higher shrinkage temperature after the experimental process remained. The shrinkage temperature of the experimental wet blue (pickled with 2% *p*TsOH) reached 108 °C, while the conventional pickled sample reached 97.9 °C. After the process, the pH of the conventional pickling solution and pH of chromed leather were lower—2.56 and 2.94 compared to a new method.

**Figure 2:**
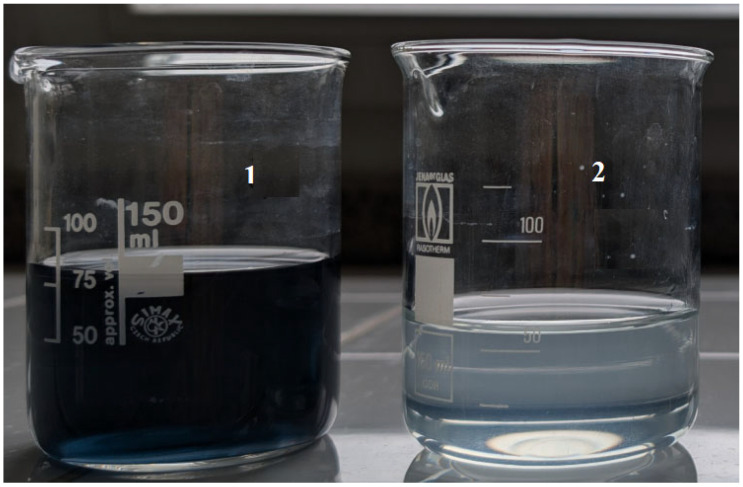
Chrome tanning solutions after the process. 1—chrome tanning after control pickling; 2—after pickling with 2% *p*TsOH.

An increased shrinkage temperature might be explained not only by the fact that sulfonic acids can bond to side chains of collagen to stabilize the structure of leather [25] but also by the fact that during chrome tanning, more chromium compounds bonded to hide, which led to better chromium compound exhaustion. Pickling with *p*TsOH had a positive effect on the consumption of chromium compounds and on their amount in the semi-finished product. Accordingly, more cross-links between collagen and chromium were formed during the process, which increased the thermostability of the hide. After chrome tanning, when visually comparing the solutions collected after the process, the amount of chromium compounds in the experimental variant solution was lower than in the control (Figure 2).

The positive effect of sulfonic acids on the consumption of chromium compounds is also mentioned in other research studies. Pickling together with methanesulfonic acid, for example, demonstrated that the use of organic acid together with NaCl during pickling resulted in 95.8% chromium compound consumption during subsequent chrome tanning. It should be noted that in comparison, conventional industrial pickling with sulfuric and formic acids achieves 81.0% chromium compound consumption during chrome tanning [40]. Other studies with aromatic sulfonic acids or their synthetic products without NaCl or with reduced NaCl concentrations during pickling have also demonstrated the advantages over conventional chrome tanning. Studies indicate that the use of different sulfonic aromatic acids improves chromium compound exhaustion compared to conventional processing [24,25,41].

The improved exhaustion of chromium compounds can be explained in that aromatic sulfonic acids provide favorable conditions for the binding of large amounts of chromium salts. These acids can bind to the structural elements of the hide and also react well with chromium salts. Theoretically, it is believed that chromium complexes are masked by sulfonic groups, which greatly improves the penetration of chromium compounds into the inner layers of the hide and prevents the formation of excessive cross-links between chromium compounds and collagen on the hide surface. Sulfonic groups can form temporary coordination bonds with chromium ions, thereby reducing the reactivity of chromium with collagen, and during further chrome tanning, when the pH of the solution changes, the coordination bonds break down and the chromium ions react with collagen [25].

**Figure 3:**
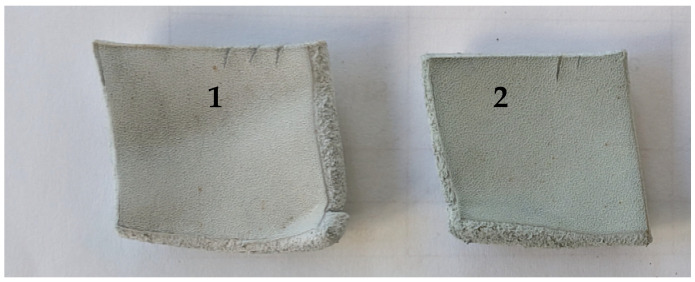
Leather samples after chrome tanning: 1—chromed after conventional pickling, 2—chromed after pickling by using 2% *p*TsOH.

After experimental pickling with *p*TsOH and chrome tanning, a wet blue that contained significantly more chromium compounds than the control chromed leather was obtained. This was also noted when comparing wet blue samples’ appearances (Figure 3). This means that to achieve the same amount of chromium compounds in wet blue as in conventional processed leather, less chromium salts could be used during chrome tanning, thus further reducing the amount of chromium compounds remaining in the wastewater after processing.

However, a darker color could also indicate greater chromium compound fixation on the surface. Therefore, in order to evaluate the effectiveness of the process, it was necessary to determine not only the total amount of chromium compounds in the samples but also their distribution in the layers (grain, middle, and flesh). To assess this, after dehydration with acetone, the total amount of chromium compounds was first determined (Table 5), then wet blue layers were separated, and the amount of chromium compounds in them was determined (Table 6). As expected, the control sample contained the lowest amount of chromium compounds, but their distribution in the layers was slightly more even than in the other sample. In general, the middle layers of all samples contained the least amount of chromium compounds, as it takes much longer for chromium compounds to penetrate the hide and distribute evenly throughout the volume. During leather processing, it is difficult to achieve a very even distribution of chromium compounds in the leather [42].

After this, wet blue mechanical properties were determined as well (Table 7). The results indicated that the samples processed using the new pickling method had greater strength (15.67 N/mm^2^). Their higher strength can be explained by the higher amount of chromium compounds in the leather. Although the distribution of these compounds in the experimental samples was slightly worse than in the control sample, their total amount was significantly higher, which contributed to the relatively high strength of the grain layer of these samples. The samples that were pickled with *p*TsOH were also more elastic (relative elongation of pickled with *p*TsOH—70.69%,) than the control sample (69.36%).

On the other hand, although the sample treated with *p*TsOH was the strongest in terms of tensile strength, its Young’s modulus was higher (40.73 N/mm^2^) compared to the conventionally treated sample (35.84 N/mm^2^). It is known that crust leather has a wide range of Young’s moduli, typically varying from 20 to 100 N/mm^2^ [43]. In general, harder leathers are associated with higher Young’s moduli. [44]. However, in this study, the experimental sample’s relative elongation at 10 N/mm^2^ was higher than that of the control sample. This suggest that, if leather is under a relatively small load, it should not be considered tough.

The FTIR analysis of the chrome-tanned samples again did not show any observable differences between leather samples conventionally pickled and pickled with *p*TsOH (Figure S2).

**Figure 4:**
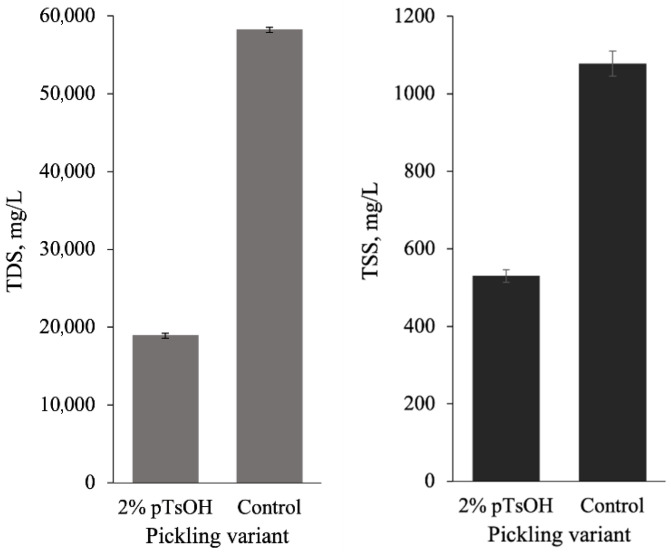
TDS and TSS in chroming solutions using different pickling methods.

After chrome tanning, as after pickling, the wastewater was collected, and the amounts of TSS and TDS were determined (Figure 4). The findings showed that after experimental treatment, TSS and TDS in the solutions were much lower than in chrome tanning solutions after conventional pickling. In control wastewater, there were twice as many TSS as after pickling with *p*TsOH. TDS in the effluents also showed that the experimental treatment had an advantage over the conventional one. It was found that TDS in the control chrome-tanning solution was 3.1 times higher than in the experimental one. These results can be explained by the additional materials used during conventional pickling (NaCl, NaCOOH). In addition, the exhaustion of chromium compounds was much lower than during conventional leather processing.

After chrome tanning, further wet finishing is performed. In order to fully evaluate the application of the new technology, it is necessary to investigate how pickling affected the subsequent processes: dyeing and fat-liquoring. After wet blue neutralization, the semi-finished product was dyed, and at the end of the process, the solutions were collected to determine the dye consumption. After fat-liquoring, part of the obtained product was used to evaluate dye penetration, while the remaining leather was dehydrated and the amount of matter soluble in dichloromethane was determined. All results are presented in Table 8.

The amount of dichloromethane-soluble (fat-liquoring) materials showed that more of the fat-liquoring substances were found in conventionally processed leather. The hide that was pickled using only *p*TsOH contained slightly fewer fat-soluble materials than the control sample. One of the factors that determined the lower fat content could have been the fact that there were many more chromium compounds in the experimental samples. During chrome tanning, when chromium compounds bind to collagen, the number of active groups capable of reacting with fat-liquoring materials decreases, so at the end of the process, there were fewer dichloromethane-soluble substances in the leather.

Dyeing exhaustion and the depth of dye penetration also showed an advantage for conventional pickling; however, the differences in dye exhaustion between samples were minimal. The slightly lower exhaustion in the experimental sample can be explained similarly to fat-liquoring: the higher chromium content in the leather reduced the number of available active sites in the wet blue that could bind with dye molecules. These results might change if lower amounts of chromium compounds are used during chrome tanning.

The depth of dye penetration indicated that in both samples, dye diffused better through the flesh layer. This trend is often observed in leather processing because the flesh layer has a more porous and open structure, which helps the dye penetrate the leather more easily [45]. Optical microscopy images of the dyed samples are presented in the Supplementary Material (Figure S3). The observation of images suggests that both samples had a qualitative grain surface without visible damage. The experimental sample exhibited a slightly more intense color.

## 4. Conclusions

Summarizing the results obtained during this study, it can be stated that the new pickling method could replace conventional pickling. The new technology does not use sodium chloride, formic acid, or sulfuric acid. The most suitable *p*TsOH amount for pickling was 2%. The pickled hide obtained after experimental pickling showed a higher shrinkage temperature, and the collected solutions contained lower amounts of TDS, TSS, and chlorides.

The chrome-tanning process further demonstrated the advantages of the new technology. During this process, the exhaustion of chromium compounds was greater than 98%, while after conventional pickling, it reached only 68.7%. After experimental processing, the amount of chromium compounds in the leather was much higher than in the control sample. This suggests that smaller amounts of basic chromium sulfate could be used during chrome tanning to achieve the same chromium content in the semi-finished product. In addition, the experimentally processed semi-finished product showed greater strength.

Infrared spectroscopy analysis of the pickled (conventionally or with 2% *p*TsOH) and chrome-tanned hide samples has not shown any observable differences in the sample structure. The tensile strength and relative elongation of chromed leather were very close independently of the used pickling method.

However, after wet finishing, the amounts of fat-liquoring materials and dye exhaustion were higher in the control samples. This should be considered in further research aimed at improving the new technology.

## Figures and Tables

**Table 1:** Technological processes.

Process	Materials *, %	Duration	Temperature, °C
A part			
Washing	H_2_O—200	1 h	19–21
Soaking	H_2_O—160, Na_2_CO_3_—1.4, Na_2_SiF_6_—0.1	14 h	19–21
Washing	H_2_O—100	20 min	19–21
Dehairing–liming	(1) H_2_O—100, Ca(OH)_2_—2.3, Na_2_S—2;	(1) 1 h	19–21
	(2) Ca(OH)_2_—2.3;	(2) 1 h	
	(3) H_2_O—100	(3) 22 h	
B part			
Washing	(1) H_2_O—200;	(1) 20 min	(1) 19–21
	(2) H_2_O—200	(2) 20 min	(2) 35–37
Deliming–bating	(1) H_2_O—40, (NH_4_)_2_SO_4_—2;	(1) 30 min	35–37
	(2) (NH_4_)_2_SO_4_—2;	(2) 30 min	
	(3) H_2_O—100, Oropon ON2—0.15	(3) 1 h	
Washing	(1) H_2_O—200;	(1) 20 min	19–21
	(2) H_2_O—200	(2) 20 min	
C part			
Pickling (experimental)	(1) H_2_O—50, *p*TsOH **	(1) 15 min	19–21
	(2) *p*TsOH **	(2) 4 h	
Pickling (control)	(1) H_2_O—50, NaCl—5.5;	(1) 15 min	
	(2) HCOONa—1;	(2) 15 min	
	(3) H_2_SO_4_—0.5;	(3) 15 min	
	(4) H_2_SO_4_—0.5;	(4) 15 min	
	(5) H_2_SO_4_—0.5;	(5) 4.5 h	
Chrome tanning (in pickling solution)	(1) Chromal—6;	(1) 4.5 h	(1) 19–21
	(2) Neutragene MG–120—0.35;	(2) 14 h	(2) 19–21
	(3) H_2_O—100	(3) 2 h	(3) 38–40
D part			
Washing	H_2_O—100	30 min	33–35
Neutralization	(1) H_2_O—150, NaHCO_3_—1.5;	(1) 30 min	
	(2) HCOONa—2	(2) 90 min	
Washing	(1) H_2_O—150	(1) 30 min	(1) 35–40
	(2) H_2_O—150	(2) 30 min	(2) 55–60
Dyeing	H_2_O—200, Sellaset Rot (H)—2	1 h	55–60
Fat-liquoring	(1) Oleal 146—2; Oleal 1946—4; Fospholiker 661—3; Fospholiker 6146—4	90	
	(2) HCOOH—1	30	
Washing	H_2_O—200;	15	30
Retanning	H_2_O—100; Mimosa tannins—2; Quebracho tannins—2	60	30
Washing	H_2_O—100;	15	30

* % based raw hide (A part), unhaired hide (B and C parts), chromed leather (D part) mass. ** *p*TsOH 0.25–1.5%.

**Table 2:** Hide pickling variants and parameters.

Variant	Materials, % Based on Hide Mass	Process Duration
1	(1) H_2_O—50, *p*TsOH—0.25	(1) 15 min
	(2) *p*TsOH—0.25	(2) 4 h
2	(1) H_2_O—50, *p*TsOH—0.5	(1) 15 min
	(2) *p*TsOH—0.5	(2) 4 h
3	(1) H_2_O—50, *p*TsOH—0.75	(1) 15 min
	(2) *p*TsOH—0.75	(2) 4 h
4	(1) H_2_O—50, *p*TsOH—1.0	(1) 15 min
	(2) *p*TsOH—1.0	(2) 4 h
5	(1) H_2_O—50, *p*TsOH—1.25	(1) 15 min
	(2) *p*TsOH—1.25	(2) 4 h
6	(1) H_2_O—50, *p*TsOH—1.5	(1) 15 min
	(2) *p*TsOH—1.5	(2) 4 h
7 (Control)	(1) H_2_O—50, NaCl—5.5;	(1) 15 min
	(2) HCOONa—1;	(2) 15 min
	(3) H_2_SO_4_—0.5;	(3) 15 min
	(4) H_2_SO_4_—0.5;	(4) 15 min
	(5) H_2_SO_4_—0.5;	(5) 4.5 h

**Table 3:** pH of pickling solutions.

Variant	pH				
	After 1st Addition of Acid	15 min After 1st Addition of Acid	After 2nd Addition of Acid	1 h After 2nd * Addition of Acid	At the End of Pickling
1	0.95 ± 0.10	7.41 ± 0.15	4.50 ± 0.20	7.28 ± 0.30	7.87 ± 0.20
2	0.98 ± 0.07	7.20 ± 0.2	1.21 ± 0.07	6.45 ± 0.10	7.27 ± 0.02
3	0.55 ± 0.01	6.11 ± 0.1	0.78 ± 0.02	2.63 ± 0.10	4.20 ± 0.02
4	0.31 ± 0.02	2.17 ± 0.02	0.61 ± 0.02	1.95 ± 0.15	2.41 ± 0.05
5	0.28 ± 0.03	2.15 ± 0.02	0.41 ± 0.01	1.85 ± 0.08	2.45 ± 0.10
6	0.09 ± 0.03	1.50 ± 0.02	0.33 ± 0.01	1.25 ± 0.05	2.10 ± 0.05
7 (Control)	0.01 ± 0.00	0.66 ± 0.05	0.13 ± 0.02	0.97 ± 0.04	2.24 ± 0.10

* During the control pickling process, acid was added three times, and the pH was measured one hour after the third addition of acid.

**Table 4:** Qualitative parameters of the pickled hide.

**Variant**	**Indexes**			
	**Shrinkage** **Temperature, °C**	**pH of Pickled Hide**	**Amount of Removed Collagen, g/kg of Hide**	**Hide Porosity, %**
1	62.1 ± 0.97	6.87 ± 0.15	0.061 ± 0.001	58.3 ± 1.2
2	62.5 ± 0.50	7.39 ± 0.20	0.073 ± 0.002	65.7 ± 2.1
3	61.3 ± 0.77	6.20 ± 0.10	0.058 ± 0.001	61.4 ± 0.7
4	58.0 ± 0.50	2.95 ± 0.02	0.065 ± 0.002	64.2 ± 0.5
5	55.0 ± 0.50	2.95 ± 0.02	-	-
6	51.3 ± 1.23	1.93 ± 0.02	-	-
7 (Control)	53.0 ± 0.50	2.28 ± 0.10	0.051 ± 0.002	67.1 ± 1.1

During the control pickling process, acid was added three times, and the pH was measured one hour after the third addition of acid.

**Table 5:** Qualitative parameters of chrome tanning process and wet blue.

Pickling Variant	Indexes				
	Shrinkage Temperature, °C	pH of Wet Blue	pH of Chrome Tanning Solution	Chromium Exhaustion, %	Cr_2_O_3_ Amount in Leather, %
2% *p*TsOH	108.0 ± 0.50	3.09 ± 0.15	2.88 ± 0.20	98.37 ± 0.85	4.73 ± 0.16
Control	97.9 ± 0.87	2.94 ± 0.2	2.56 ± 0.07	68.7 ± 1.30	3.52 ± 0.14

**Table 6:** Chromium compounds in wet blue, depending on the pickling.

Pickling Variant	Cr_2_O_3_ Amount in Wet Blue, %		
	Grain Layer	Middle Layer	Flesh Layer
2% *p*TsOH	5.42 ± 0.18	3.80 ± 0.06	5.70 ± 0.24
Control	3.53 ± 0.08	3.00 ± 0.10	3.41 ± 0.10

**Table 7:** Chrome-tanned leather physical and mechanical properties.

Pickling Variant	Indexes			
	Tensile Strength of Leather, N/mm^2^	Relative Elongation of Leather, %	Relative Elongation of Leather at the Strain 10 N/mm^2^, %	Young’s Modulus, N/mm^2^
2% *p*TsOH	15.67 ± 0.61	70.69 ± 1.52	23.96 ± 1.07	40.73 ± 1.88
Control	14.35 ± 0.58	69.36 ± 2.02	23.61 ± 1.11	35.84 ± 1.71

**Table 8:** Pickling effect on wet-finishing.

Pickling Variant	Indexes			
	Amount of Matter Soluble in Dichloromethane, %	Dye Exhaustion, %	Depth of Dye Penetration into the Leather, %	
			Grain Layer	Flesh Layer
2% *p*TsOH	6.36 ± 0.15	82.38 ± 1.40	8.28 ± 0.38	9.14 ± 0.21
Control	6.91 ± 0.24	84.10 ± 1.30	8.11 ± 0.33	12.12 ± 0.54

## Data Availability

The original contributions presented in this study are included in the article/Supplementary Material. Further inquiries can be directed to the corresponding author.

## Supplementary Materials

The following supporting information can be downloaded at https://www.mdpi.com/article/10.3390/ma19030471/s1, Figure S1: FTIR spectra of hide samples after pickling: 1—control (pickled conventionally); 2—experimental (pickled with 2% *p*TsOH). Figure S2: FTIR spectra of leather samples after chrome tanning: 1—control (pickled conventionally); 2—experimental (pickled with 2% *p*TsOH). Figure S3: optical microscope images (magnification 24 times) of crust leather: A—experimental (pickled with 2% *p*TsOH) and B—control (pickled conventionally). Table S1: Data of tensile test of control (pickled conventionally) and experimental (pickled with 2% *p*TsOH) chrome-tanned leather.

## Abbreviations

The following abbreviations are used in this manuscript:

*p*TsOH	*p*-Toluenesulfonic acid monohydrate
TDS	Total dissolved solids
TSS	Total suspended solids
