# Supplementary material for: Salt-Free Pickling with Sulfonic Acid as an Approach to Cleaner Leather Processing
Source: Materials (Basel). 2026 Jan 24;19(3):471. doi: 10.3390/ma19030471 (PMC12898438; doi:10.3390/ma19030471)
Supplement: Supplementary file 1 [file materials-19-00471-s001.zip › materials-4054596-supplementary.pdf]

materials

Supporting information

Salt free pickling with sulfonic acid as an approach to cleaner leather processing

Renata Biškauskaitė-Ulinskė and Virgilijus Valeika *

Faculty of Chemical Technology, Kaunas University of Technology, Radvilenu pl. 19, 50254 Kaunas, Lithuania
* Correspondence: virgilijus.valeika@ktu.lt;

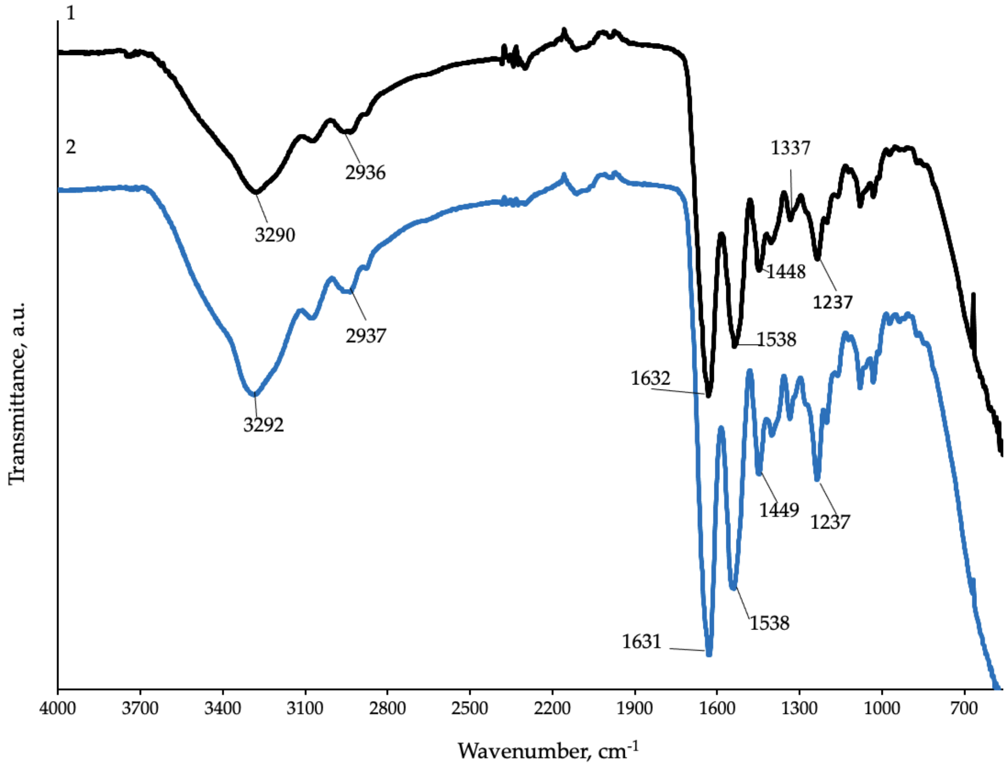

Figure S1. FTIR spectra of hide samples after pickling. 1 – control (pickled conventionally); 2 – experimental (pickled with 2% *p*TsOH).

 https://doi.org/10.3390/ma19030471

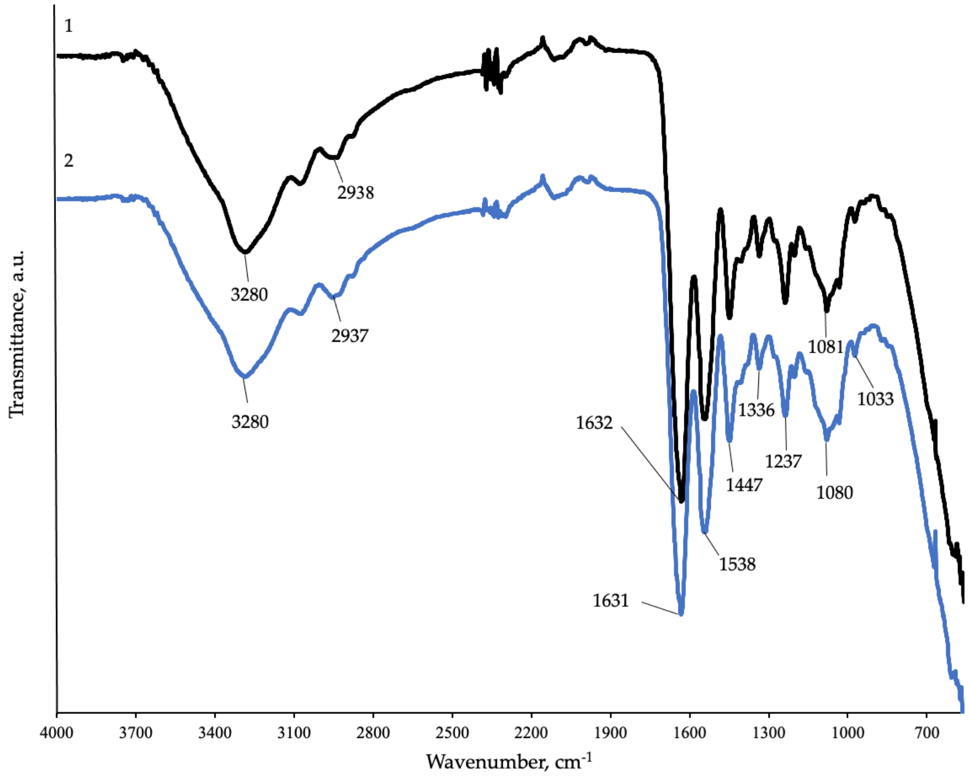

Figure S2. FTIR spectra of leather samples after chrome tanning. 1 – control (pickled conventionally); 2 – experimental (pickled with 2% *p*TsOH).

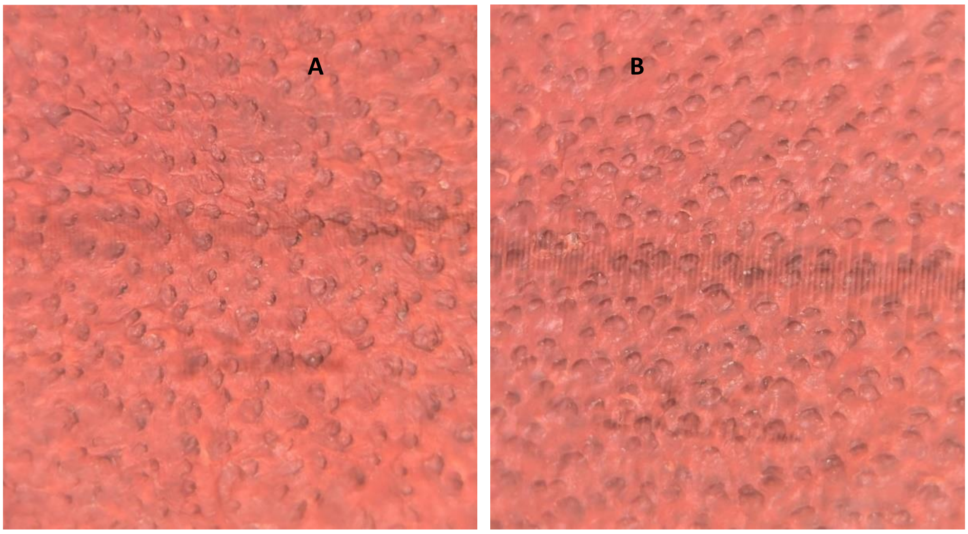

Figure S3. Optical microscope images (magnification 24 times) of crust leather: A – experimental (pickled with 2% *p*TsOH) and B – control (pickled conventionally).

Table S1. Data of tensile test of control (pickled conventionally) and experimental (pickled with 2% *p*TsOH) chrome tanned leather.

Test Name : control
Test Type : Tensile
Test Speed : 100.000 mm/min
Pretension : 5.000 N
Width : 10.000 mm

Comments: 1, 2, 3 – samples parallel; 4, 5, 6 - perpendicular to backbone.

Test No	Stress @ Break (MPa)	Elong. @ Break (mm)	Youngs Modulus (MPa)	Sample Length (mm)	Thickness (mm)	Force @ Break (N)	Elong. @ Yield (mm)
1	15.296	37.844	29.145	50.096	3.668	561.057	37.557
2	14.746	34.518	35.756	50.107	3.798	560.053	33.944
3	15.336	35.423	40.602	50.078	3.700	567.432	34.704
4	13.067	34.928	32.823	50.048	4.526	591.412	34.205
5	13.573	29.598	39.551	50.092	4.520	613.500	29.167
6	14.088	35.775	37.152	50.067	4.658	656.219	35.244
Min	13.067	29.598	29.145	50.048	3.668	560.053	29.167
**Mean**	**14.351**	**34.681**	**35.838**	**50.081**	**4.145**	**591.612**	**34.137**
Max	15.336	37.844	40.602	50.107	4.658	656.219	37.557

Test Name : experimental
Test Type : Tensile
Test Speed : 100.000 mm/min
Pretension : 5.000 N
Width : 10.000 mm

Comments: samples 1, 2, 3 - parallel; 4, 5, 6 - perpendicular to backbone.

Test No	Stress @ Break (MPa)	Elong. @ Break (mm)	Youngs Modulus (MPa)	Sample Length (mm)	Thickness (mm)	Force @ Break (N)	Elong. @ Yield (mm)
1	16.786	34.676	44.551	50.068	4.216	707.698	34.150
2	16.373	37.347	39.068	50.071	4.162	681.444	36.437
3	16.202	36.207	44.934	50.057	4.100	664.282	35.441
4	15.188	37.968	37.377	50.084	4.112	624.530	37.342
5	15.445	33.813	41.315	50.062	4.070	628.615	27.208
6	14.020	32.045	37.159	50.075	4.000	560.800	31.758
Min	14.020	32.045	37.159	50.057	4.000	560.800	27.208
**Mean**	**15.669**	**35.343**	**40.734**	**50.070**	**4.243**	**644.562**	**33.723**
Max	16.786	37.968	44.934	50.084	4.216	707.698	37.342

https://doi.org/10.3390/ma19030471
